# GluN2B-containing NMDA receptor attenuated neuronal apoptosis in the mouse model of HIBD through inhibiting endoplasmic reticulum stress-activated PERK/eIF2α signaling pathway

**DOI:** 10.3389/fnmol.2024.1375843

**Published:** 2024-04-04

**Authors:** Mengxue Wu, Shilian Xu, Kai Mi, Shuang Yang, Yuanyuan Xu, Jie Li, Junyang Chen, Xiaomin Zhang

**Affiliations:** Department of Physiology, School of Basic Medicine, Kunming Medical University, Kunming, Yunnan, China

**Keywords:** hypoxic–ischemic brain damage, GluN2B, ER stress, UPR, PERK

## Abstract

**Introduction:**

Neonatal hypoxic-ischemic brain damage (HIBD) refers to brain damage in newborns caused by hypoxia and reduced or even stopped cerebral blood flow during the perinatal period. Currently, there are no targeted treatments for neonatal ischemic hypoxic brain damage, primarily due to the incomplete understanding of its pathophysiological mechanisms. Especially, the role of NMDA receptors is less studied in HIBD. Therefore, this study explored the molecular mechanism of endogenous protection mediated by GluN2B-NMDAR in HIBD.

**Method:**

Hypoxic ischemia was induced in mice aged 9-11 days. The brain damage was examined by Nissl staining and HE staining, while neuronal apoptosis was examined by Hoechst staining and TTC staining. And cognitive deficiency of mice was examined by various behavior tests including Barnes Maze, Three Chamber Social Interaction Test and Elevated Plus Maze. The activation of ER stress signaling pathways were evaluated by Western blot.

**Results:**

We found that after HIBD induction, the activation of GluN2B-NMDAR attenuated neuronal apoptosis and brain damage. Meanwhile, the ER stress PERK/eIF2α signaling pathway was activated in a time-dependent manner after HIBE. Furthermore, after selective inhibiting GluN2B-NMDAR in HIBD mice with ifenprodil, the PERK/eIF2α signaling pathway remains continuously activated, leading to neuronal apoptosis, morphological brain damage. and aggravating deficits in spatial memory, cognition, and social abilities in adult mice.

**Discussion:**

The results of this study indicate that, unlike its role in adult brain damage, GluN2B in early development plays a neuroprotective role in HIBD by inhibiting excessive activation of the PERK/eIF2α signaling pathway. This study provides theoretical support for the clinical development of targeted drugs or treatment methods for HIBD.

## 1 Introduction

Neonatal hypoxic–ischemic brain damage (HIBD) refers to neonatal brain damage caused by hypoxia and reduced or even halted cerebral blood flow during the perinatal period. HIBD is a leading cause of neonatal death and chronic neurological sequelae (Nabetani et al., 2022). Approximately 48% of HIBD survivors suffer from varying degrees of brain damage, with long-term potential impacts on their schooling and socioeconomic productivity (Lee et al., 2013). Moreover, HIBD is the primary cause of epileptic seizures in children (accounting for about 38% of epilepsy cases), where high epilepsy burden is a significant risk factor for death and neurological examination anomalies (Glass et al., 2016). Currently, the clinical treatment strategy for HIBD primarily involves hypothermia therapy, but due to its narrow therapeutic window and the lack of early diagnostic indicators for HIBD, the optimal treatment period is often missed (Jia et al., 2018). Thus, there is an urgent need to study its pathogenesis to find more suitable therapeutic targets for adjunctive treatment.

N-methyl-D-aspartate receptors (NMDARs) are excitatory glutamate receptors widely present in the central nervous system. They play a complex role in integrating and converting information by detecting extracellular glutamate signals and transforming them into different intracellular signal outputs, thus participating in the normal growth of neurons and axons, brain development, and maturation (Cull-Candy et al., 2001; Lai et al., 2014; Hardingham, 2019). NMDARs have a high affinity for glutamate, which is the basis for NMDAR-induced excitotoxicity (Karadottir et al., 2005). Under pathological conditions, excessive glutamate release leads to NMDAR overactivation, causing a large influx of Ca^2+^ into neurons, triggering excitotoxicity, and activating downstream death programs (Wu and Tymianski, 2018). NMDARs are heterotetrameric receptor complexes composed of four different subunits, including structural subunits GluN1 and regulatory subunits GluN2 and GluN3 (Traynelis et al., 2010). Each subunit renders the receptor unique functions and characteristics (Cull-Candy et al., 2001). NMDARs are typically composed of two GluN1 and two GluN2A-D subunits, with the GluN1/GluN2A/GluN2B complex being the main receptor in hippocampal synapses (Luo et al., 1997; Rauner and Kohr, 2011). Additionally, their expression varies during different developmental stages. In early development, the GluN2B subunit dominates, with higher expression than GluN2A while GluN2A slowly increases, peaking in middle age before declining sharply (Knox et al., 2013; Siu et al., 2017). In mature neurons, GluN2A and GluN2B have opposite roles; GluN2A-NMDAR exerts a neuroprotective effect, while GluN2B-NMDAR induces excitotoxicity leading to neuronal death upon overactivation (Chen et al., 2008). The role of GluN2B, the dominant subunit in early development, in neonatal hypoxic–ischemic brain damage remains controversial (Takagi et al., 2003; Martel et al., 2009; Lai et al., 2016). Our previous studies found that after hypoxic–ischemic (HI), GluN2A subunit expression significantly downregulates within 72 h and does not respond to hypoxic–ischemic injury. However, GluN2B, the dominant subunit in early development, exerts a neuroprotective effect by binding to PSD95, inconsistent with its role in adult brain stroke. This may be due to GluN2B-NMDAR being the dominant subunit in early development, thus being the primary bearer of neuroprotective functions (Zhang et al., 2023). However, the molecular mechanisms of the protective role of GluN2B in HIBD is still not clear yet.

The endoplasmic reticulum (ER) is the main site for translation of membrane and secretory proteins in cells, helping nascent proteins to fold and assemble correctly to function normally. ER homeostasis is fundamental for normal cellular operation. During neuronal hypoxia-ischemia, energy failure leads to an increase and accumulation of misfolded proteins. In response, the ER initiates the unfolded protein response (UPR) to maintain normal function. The UPR pathway has a broad role, involving gene transcription and mRNA translation, repairing misfolded proteins, and degrading surplus or misfolded proteins. UPR is mainly mediated by three different receptors located on the ER membrane, namely IRE1α, PERK, and ATF6α, which cooperate to maintain ER homeostasis (Hetz et al., 2020). However, excessive or prolonged activation of the UPR pathway can trigger apoptosis. Previous reports showed that the UPR pathway is activated in HIBD (Badiola et al., 2011; Carloni et al., 2014; Chavez-Valdez et al., 2016), but the outcome of this activation remains unclear. In our study, we found that GluNB-NMDAR exerts a neuroprotective effect by regulating the activity of the PERK signaling pathway in the ER stress response after HIBD. This may provide a new insights for targeted clinical treatment of HIBD.

## 2 Materials and methods

### 2.1 Animals

Adult male and female C57BL/6 J mice obtained and acclimated to the colony room for at least 2 weeks prior to mating. The room was maintained under a 14-h light/10-h dark cycle, with free access to food and water, at a controlled temperature (22°C ± 1°C) and humidity. Experimental subjects were the offspring of these pairs, with pregnant females housed separately until the pups were born. All animal experimental procedures followed the International Guiding Principles for Biomedical Research Involving Animals (1985) established by the Council for International Organizations of Medical Sciences, as well as the animal ethical guidelines of Kunming Medical University.

### 2.2 Establishment of the HIBD model

C57BL/6 pups, aged 9–11 days with an average weight of 5 g, were randomly divided into groups. The model group was created using the classic Vannucci method for inducing hypoxic–ischemic brain disease. After anesthetizing the mice by isoflurane inhalation, they were fixed on a stereotaxic operating table. Following 75% ethanol disinfection of the surgical field, an incision was made in the neck’s midline to expose the left common carotid artery (LCCA). The LCCA was ligated with 4–0 suture, tightened, and held for a few seconds to ensure complete arterial occlusion, and then the skin was sutured. The pups were allowed to recover for 1 h next to their mothers in their cages. The mice were placed in a sealed hypoxic chamber at 34°C, with nitrogen reducing oxygen concentration to 8% for 45 min, and then sent back to their mothers. The sham group underwent the same anesthetic and LCCA exposure procedure but without ligation, followed by skin suturing and return to their original cages after recovery.

### 2.3 Pharmacological intervention

The pups were divided into groups: sham, HI, HI + CCT020312, HI + ifenprodil, and HI + NaCl. CCT020312, a selective PERK/eIF2α agonist (Stockwell et al., 2012) (injection concentration of 0.4 mg/mL, dose of 2 mg/kg), was administered intraperitoneally immediately after surgery, once every 24 h for a total of three times. Ifenprodil, a specific inhibitor of GluN2B (Brittain et al., 2012) (injection concentration of 1 mg/kg, dose of 20 mg/kg), was administered intraperitoneally immediately post-modeling, with NaCl treatment following the same procedure. The brain was harvested for subsequent experiments at least two hours after drug injection.

### 2.4 TTC (2,3,5-triphenyltetrazolium chloride) staining

At 72 h post-modeling, the mice were killed and their brains were harvested and immediately frozen at −20°C for 17 min. The brains were sliced from the olfactory bulb to the cerebellum into five 1 mm-thick slices and stained in pre-warmed 1% TTC solution at 37°C for 20 min. After discarding the TTC solution, the slices were fixed in 4% paraformaldehyde for 24 h. Image J was used to analyze brain slice images and calculate the infarct area.

### 2.5 Hoechst staining

To detect the evidence of apoptosis, the brains were perfused at 72 h post-modeling, fixed in 4% paraformaldehyde for 24 h, dehydrated in an ethanol gradient, and vitrified by dimethylbenzene before being embedded in paraffin. Sections of 5 μm thickness were covered with Hoechst 33342 staining solution for 8 min in a dark environment, washed with PBS, and coverslipped. Fluorescent microscopy was used to observe and count brightly stained apoptotic cells.

### 2.6 HE / Nissl staining

Following a 48-h period post-modeling or drug administration, the brains were extracted and fixed in 4% paraformaldehyde for 24 h. Subsequently, they were rinsed under running water, dehydrated through a graded ethanol series, clarified using xylene, and then embedded in paraffin. The 5 μm sections were affixed to adhesive-coated slides, underwent staining with hematoxylin and eosin (HE) or Nissl stain following dewaxing, were dehydrated once more, clarified, and coverslipped using neutral balsam. Photomicrographs were captured utilizing a Leica upright microscope.

### 2.7 Western blot

Cortical tissues from the infarcted area were harvested at 3, 6, 24, and 48 h post-modeling and homogenized in RIPA lysis buffer containing PMSF and phosphatase inhibitors. Depending on the molecular weight of the target protein, 8% or 12% SDS-PAGE gels were used for electrophoresis, followed by wet transfer of proteins onto 0.45 mm PVDF membranes. After blocking with non-fat milk for 2 h and TBST washing, primary antibodies (PI3K, p-PI3K, Akt, p-Akt, PERK, p-PERK, eIF2α, p-eIF2α, ATF4, CHOP, Caspase3, Cleaved Caspase3, IRE1, XBP1s, ATF6, Bax, Bcl-2) were applied overnight at 4°C. Following incubation with corresponding secondary antibodies for 2 h, the membranes were exposed using an enhanced chemiluminescence solution. The bands were scanned for grayscale values using Image J software.

### 2.8 Behavioral testing

#### 2.8.1 Barnes maze

Mice from the control, HIBD, and ifenprodil groups (at least 8 in each group) were tested in the Barnes maze after growing in a normal rearing environment until 4 weeks post-modeling. Before the first training session on the first day, each mouse was placed in the target hole for 4 min for acclimatization. The mice were then transferred to a central black cylinder in the maze, and the timer was started 30 s after removing the cylinder, allowing the mice to search for the target hole independently. The timer stopped once the target hole was found, with a maximum duration of 240 s. Mice that failed to find the target hole were placed back into it for another 4 min of acclimatization before being returned to their cage. Training continued for 6 consecutive days, twice a day, with a 2-h interval between sessions. The seventh day was the test day, with only one test conducted. The time taken by the mice to find the target hole was recorded to assess potential impairments in spatial memory.

#### 2.8.2 Three-chamber social interaction test

Mice were allowed to freely explore the middle chamber for 5 min. Then, one chamber contained a novel mouse, and the other was empty. The test lasted for 10 min, and the proportion of time spent interacting with the novel mouse was recorded to determine if there were any abnormalities in social behavior.

#### 2.8.3 Elevated plus maze

Mice were placed in the center of the elevated plus maze, facing the closed arm, and timed for 10 min. The time spent in the open and closed arms was recorded to assess fear responses to the external environment.

#### 2.8.4 Motor function assessment

The corner turn test, cylinder test, negative geotaxis test, and grip strength test were used to assess motor abilities. In the corner turn test, the direction of turns was recorded 10 times in a 30° angle, with the score being the number of left turns minus right turns. In the cylinder test, the number of times the forelimbs touched the walls in a 15 cm diameter cylinder during 20 standing attempts was recorded, with the score being the number of right forelimb touches plus half the number of touches by both arms. In the negative geotaxis test, the time taken for the mouse to turn when placed head-down on a 70° slope was recorded, with five trials per mouse and the average time calculated. In the grip test, mice were scored using the Longa scoring method based on their coordination of hind limbs and tail when escaping or falling from a 1 mm wire, with five tests per mouse. The apparatus was cleaned with alcohol after each test to prevent olfactory interference.

## 3 Results

### 3.1 Significant brain damage observed post-HI

**Figure 1:**
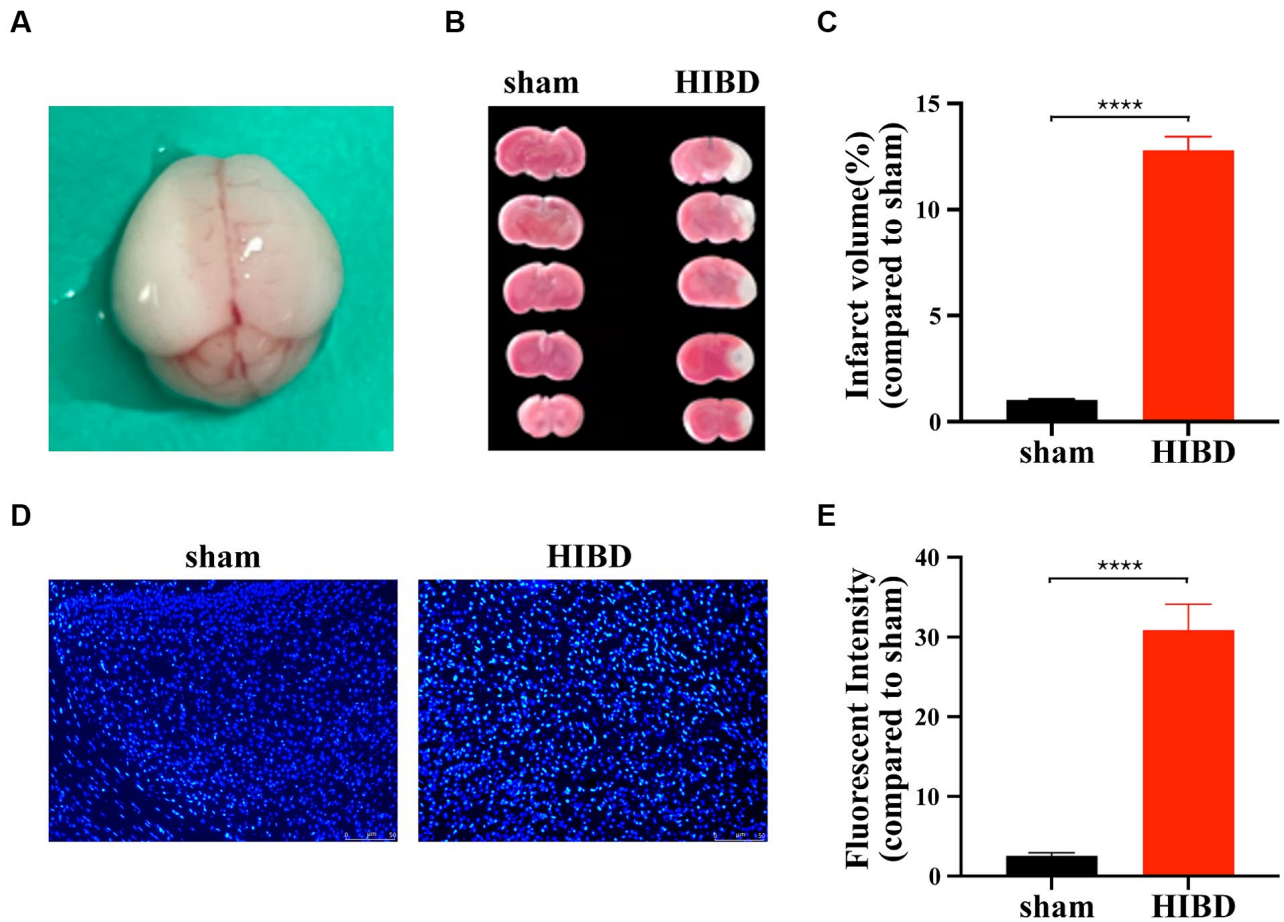
Hypoxic ischemia causes neuronal death. **(A)** Forty-eight hours after surgery, grayish-white infarction area appeared in the left hemispherecerebral. **(B)** Forty-eight hours after surgery, infarction area was detected by TTC staining for HIE and sham group mice. **(C)** The statistical analysis of TTC staining. Compared with the sham group, the infarct volume increased significantly in HIE group (^****^p < 0.0001, *n* = 12). **(D)** Coronal section of the brain was performed 48 h after surgery in HIE and sham group mice. Apoptosis of cortical neurons was detected by Hoechst staining. **(E)** The statistical analysis of Hoechst staining. Compared with the sham group, the fluorescent intensity increased significantly in HIE group. Scale bar: 50 μm. (^****^p < 0.0001, *n* = 7).

A hypoxic–ischemic brain disease (HIBD) mouse model was established in 9-11-day-old mice by ligation of the left common carotid artery combined with hypoxia. Brain harvesting after HI 72 h revealed white infarction lesions in the left brain (sham vs. HIBD: ^****^p < 0.0001, n = 12), indicating successful modeling (Figures 1A–C). Additionally, Hoechst staining showed significantly increased fluorescence intensity in brain sections of HIBD mice 48 h post-HI compared to the sham group (sham vs. HIBD: ^****^p < 0.0001, *n* = 7) (Figures 1D,E), suggesting evident neuronal apoptosis. These results indicate that our modeling method effectively induces the HIBD mouse model.

### 3.2 Activation of the PERK/eIF2α Signaling pathway post-HI

**Figure 2:**
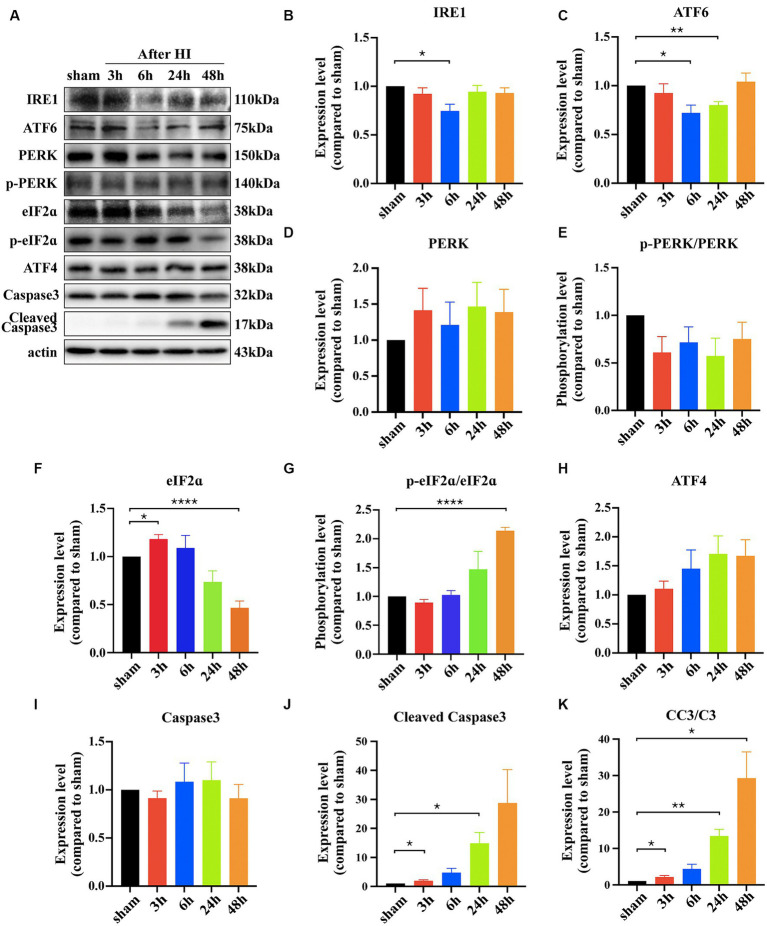
Hypoxic ischemia activates the PERK/eIF2ɑ pathway of the UPR. **(A)** The cortical proteins of sham and HIE mice after surgery 3 h, 6 h, 24 h and 48 h was extracted and measured by Western Blot to detect IRE1, ATF6, PERK, p-PERK, eIF2ɑ, p-eIF2ɑ, ATF4, Caspase3, Cleaved Caspase3 expression levels. **(B–K)** Statistical analysis of Western Blot results. Compared with the sham group, the expression level of IRE1, ATF6, eIF2ɑ, Caspase3 and Cleaved Caspase3 showed significant difference (^*^p < 0.05, ^**^p < 0.01, ^****^p < 0.0001, *n* = 3).

To verify whether the UPR signaling pathway participates in neuronal apoptosis post-HI, key protein molecules of the three pathways were examined at 3 h, 6 h, 24 h, and 48 h. IRE1 showed a transient downregulation at 6 h and then returned to normal levels (3 h vs. sham: *p* = 0.2565; 6 h vs. sham: *p = 0.0111; 24 h vs. sham: *p* = 0.4180; 48 h vs. sham: *p* = 0.2461, *n* = 4). ATF6 demonstrated a downward trend, reaching its lowest at 24 h and recovering at 48 h (3 h vs. sham: *p* = 0.4539; 6 h vs. sham: *p = 0.0138; 24 h vs. sham: **p = 0.0015; 48 h vs. sham: *p* = 0.6688, n = 4). PERK protein levels did not show significant changes (3 h vs. sham: *p* = 0.2498; 6 h vs. sham: *p* = 0.5497; 24 h vs. sham: *p* = 0.2366; 48 h vs. sham: *p* = 0.2888, *n* = 3), and neither did its phosphorylation levels (p-PERK/PERK) (3 h vs. sham: *p* = 0.0809; 6 h vs. sham: *p* = 0.1568; 24 h vs. sham: *p* = 0.0852; 48 h vs. sham: *p* = 0.2319, *n* = 3). However, downstream protein eIF2α reacted, showing a brief increase at 3 h and then a significant decrease at 48 h (3 h vs. sham: *p* = 0.0201 6 h vs. sham: *p* = 0.5343; 24 h vs. sham: *p* = 0.0829; 48 h vs. sham: ****p < 0.0001, *n* = 3), with its phosphorylation level (p-eIF2α/eIF2α) showing an increasing trend and significant difference at 48 h (3 h vs. sham: *p* = 0.1202; 6 h vs. sham: *p* = 0.7440; 24 h vs. sham: *p* = 0.1993; 48 h vs. sham: ****p < 0.0001, *n* = 3). ATF4 showed no significant changes (3 h vs. sham: *p* = 0.4689; 6 h vs. sham: *p* = 0.2350; 24 h vs. sham: *p* = 0.0857; 48 h vs. sham: *p* = 0.0747, *n* = 3). Cleaved Caspase3 was used as an apoptosis marker to detect neuronal death, its expression level increased significantly after HI 24 h (3 h vs. sham: *p = 0.0399 6 h vs. sham: *p* = 0.057; 24 h vs. sham: **p = 0.0025; 48 h vs. sham: *p = 0.0169, *n* = 3)(Figures 2A–K).

### 3.3 Ifenprodil inhibits overactivation of the PERK-eIF2α signaling pathway

**Figure 3:**
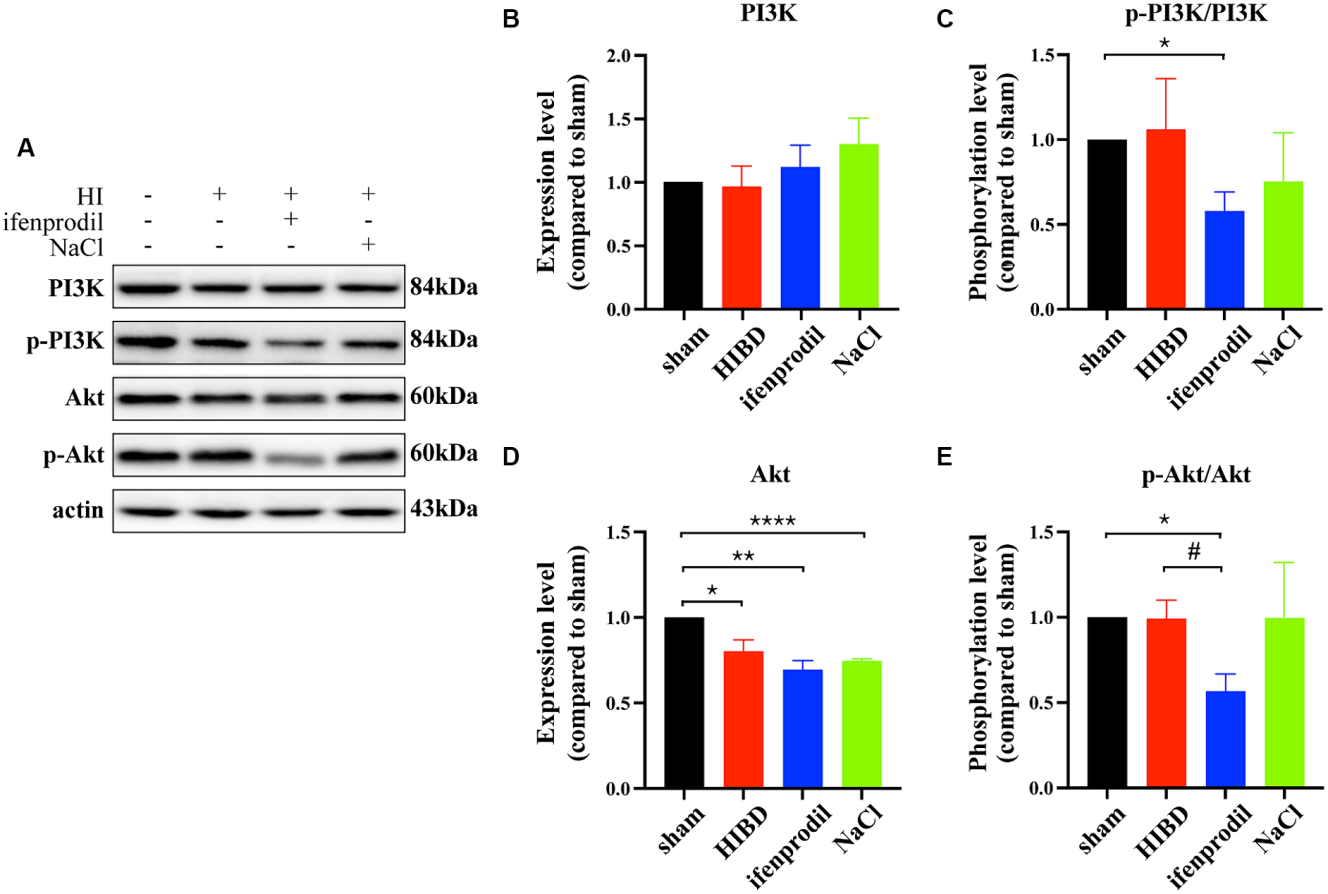
Ifenprodil inhibits the phosphorylation level of PI3K/Akt signal pathway. **(A)** Ifenprodil, the specific inhibitor of GluN2B, was administered intraperitoneally immediately after surgery (20 mg/kg). The cortical protein of sham, HIE, ifenprodil and NaCl mice of the same age was extracted and measured by Western Blot to detect PI3K, p-PI3K, Akt, p-Akt expression levels. **(B-E)** Statistical analysis of **(A)**. ^*^indicates a significant difference compared to the sham group (^*^p < 0.05, ^**^p < 0.01, *n* = 3); #indicates a difference compared to HIE group (# *p* < 0.05, ^****^p < 0.0001, *n* = 3).

**Figure 4:**
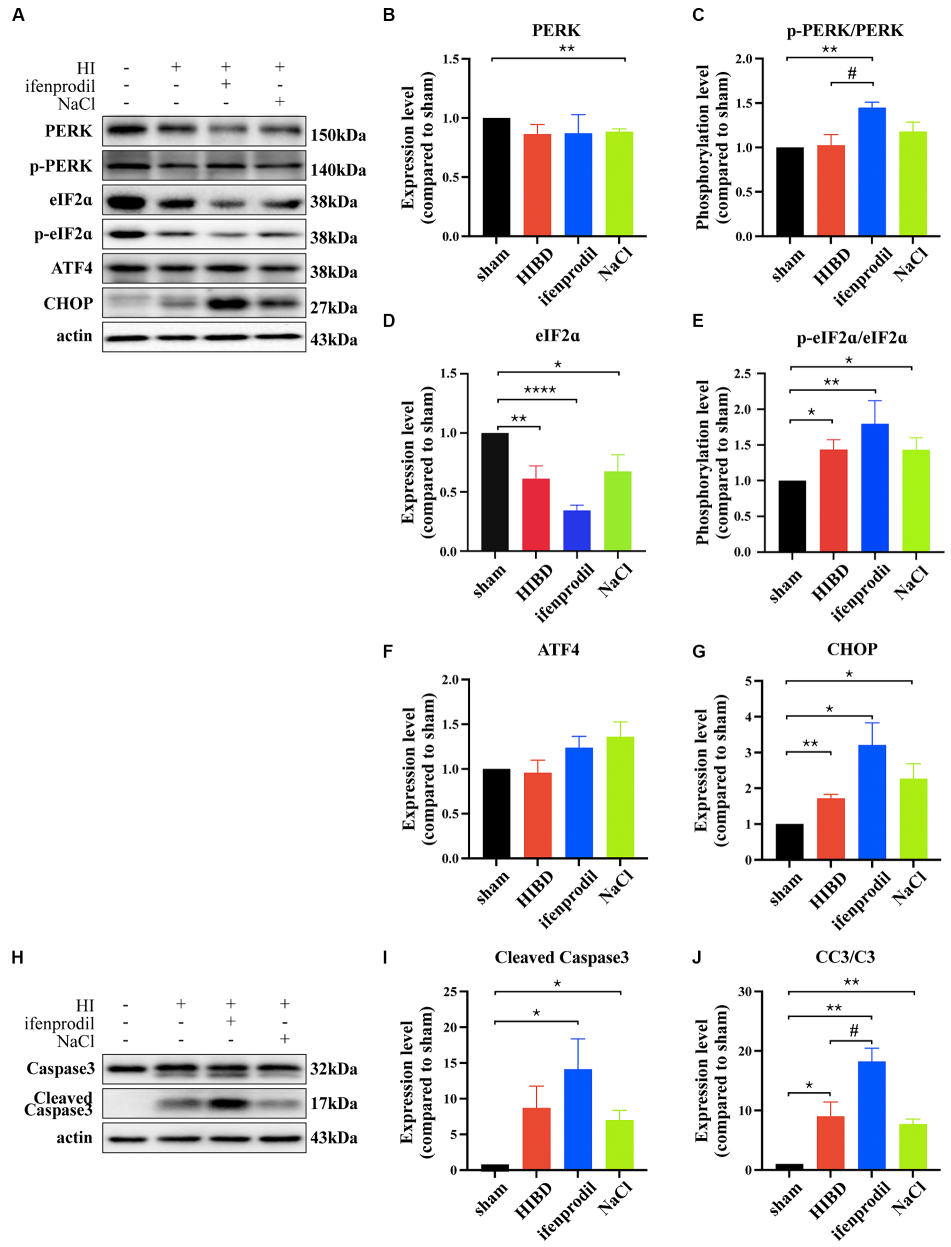
GluN2B inhibits the overactivition of PERK/eIF2ɑ pathway. **(A)** Ifenprodil, the specific inhibitor of GluN2B, was administered intraperitoneally immediately after surgery (20 mg/kg). The cortical protein of sham, HIE, ifenprodil and NaCl mice of the same age was extracted and measured by Western Blot to detect PERK, p-PERK, eIF2ɑ, p-eIF2ɑ, ATF4, CHOP expression levels. **(B-E)** Statistical analysis of **(A)**. *indicates that compared to the sham group, Differences (^*^p < 0.05, ^**^p < 0.01, ^***^p < 0.001, ^****^p < 0.0001, *n* = 3); #indicates a difference compared to HIE group (#*p* < 0.05, *n* = 3). **(H)** Ifenprodil, the specific inhibitor of GluN2B, was administered intraperitoneally immediately after surgery (20 mg/kg). The cortical protein of sham, HIE, ifenprodil and NaCl group mice of the same age was extracted and measured by Western Blot to detect Caspase3, Cleaved Caspase3 expression levels. **(I–J)** Statistical analysis of **(H)**. *indicates a significant difference compared to the sham group (^*^p < 0.05, ^**^p < 0.01, *n* = 3); #indicates a difference compared to HIE group (#*p* < 0.05, *n* = 3).

**Figure 5:**
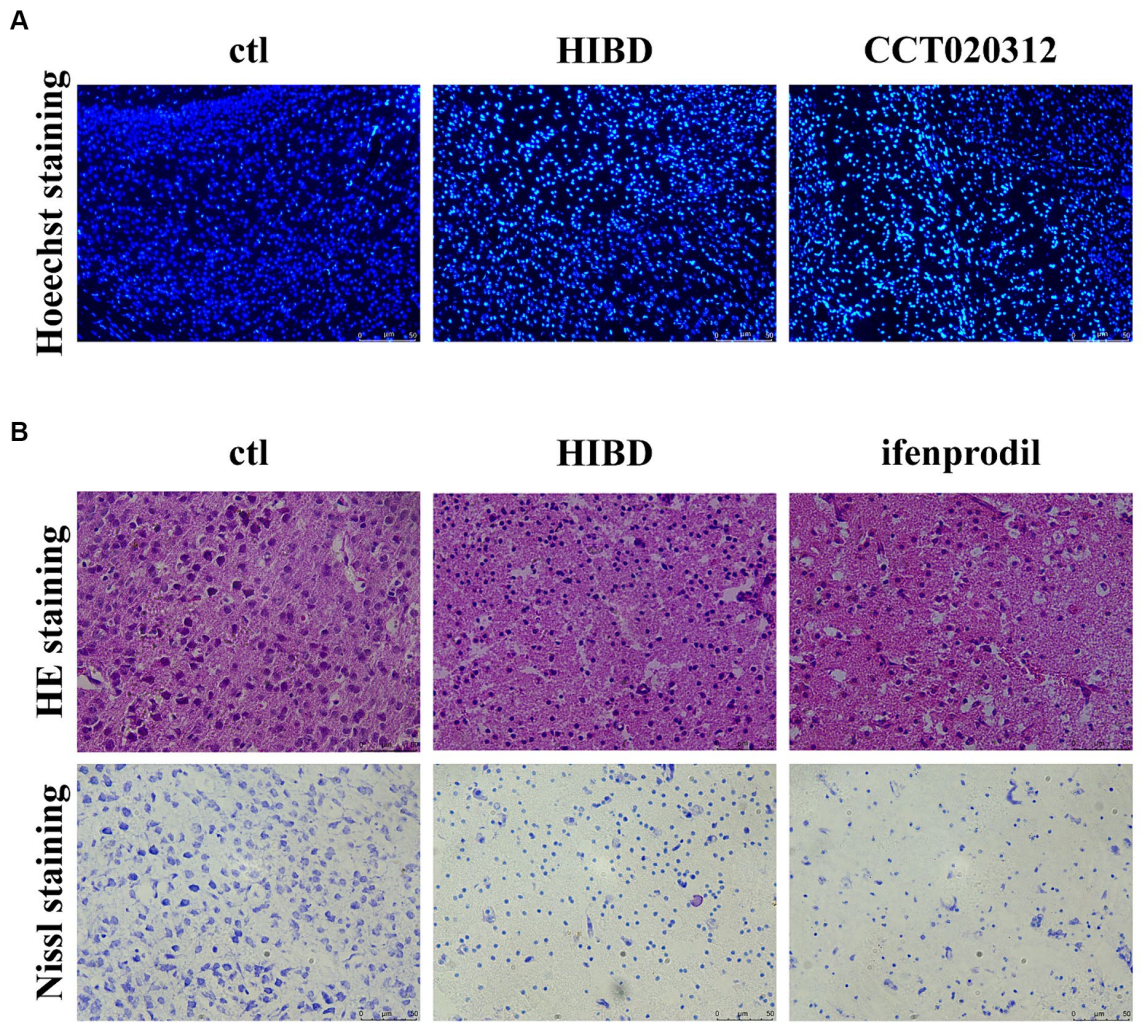
Ifenprodil exacerbates tissue pathology and neuronal apoptosis. **(A)** PERK/eIF2ɑ selected agonist CCT020312 was administered intraperitoneally immediately after surgery, once every 24 h for a total of three times (2 mg/kg). Coronal section of the brain was performed 48 h after surgery in sham, HIE and CCT020312 group mice. Apoptosis of cortical neurons was detected by Hoechst staining. Scale bar: 50 μm. **(B)** Coronal section of the brain was performed 48 h after surgery in sham, HIE and ifenprodil group mice. HE staining and Nissl staining was used to detect the extent of tissue pathology and neuronal apoptosis. Scale bar: 50 μm.

Ifenprodil is a selective inhibitor of the GluN2B receptor (Brittain et al., 2012). Western blot analysis revealed how ifenprodil affects downstream responses. First, changes in the PI3K/Akt signaling pathway were observed, with significant inhibition of PI3K phosphorylation post-ifenprodil administration (ifenprodil vs. sham: *p = 0.0204; HIBD vs. sham, *p* = 0.8488; HIBD vs. ifenprodil, *p* = 0.2063, *n* = 3). Akt expression was suppressed (ifenprodil vs. sham: **p = 0.0046; HIBD vs. sham, *p = 0.0413; HIBD vs. ifenprodil, *p* = 0.2764; NaCl vs. sham: ****p < 0.0001, *n* = 3), and its phosphorylation level was also downregulated compared to the HIBD group (ifenprodil vs. sham: *p = 0.0126; HIBD vs. sham, *p* = 0.9406; HIBD vs. ifenprodil, #*p* = 0.0462, *n* = 3), indicating further inhibition of PI3K/Akt signal pathway’s protective role (Figures 3A–E), consistent with our previous findings. Secondly, ifenprodil raised PERK phosphorylation levels (ifenprodil vs. sham: **p = 0.0023; HIBD vs. sham, *p* = 0.8468; HIBD vs. ifenprodil, #*p* = 0.0355, *n* = 3). The expression of total eIF2α protein was suppressed by HIBD (ifenprodil vs. sham: ****p < 0.0001; HIBD vs. sham, **p = 0.0495; HIBD vs. Ifenprodil: *p* = 0.0632; NaCl vs. sham, *p = 0.0332, *n* = 4), but its phosphorylation level was increased (ifenprodil vs. sham: **p = 0.0040; HIBD vs. sham, *p = 0.0240; HIBD vs. ifenprodil, *p* = 0.1890, *n* = 4). Although there were no significant changes in downstream ATF4 (ifenprodil vs. sham: *p* = 0.1344; HIBD vs. sham, *p* = 0.7757; HIBD vs. ifenprodil, *p* = 0.2128, *n* = 3), the pro-apoptotic factor CHOP was increased (ifenprodil vs. sham: *p = 0.0231; HIBD vs. sham, **p = 0.0031; HIBD vs. ifenprodi, *p* = 0.0762; NaCl vs. sham, *p = 0.0387, *n* = 3), suggesting that inhibition of GluN2B by ifenprodil keeps the PERK signaling pathway in a persistently overactivated state (Figures 4A–G). The consequences of this continuous activation are shown in Figures 4H–J, with higher expression levels of cleaved caspase3 than in the HIBD group (cleaved caspase3 expression level (ifenprodil vs. sham: *p = 0.0355; HIBD vs. sham, *p* = 0.0665; HIBD vs. ifenprodil, *p* = 0.3548; NaCl vs. sham, *p = 0.011, *n* = 3) and the ratio of cleaved caspase3/caspase3 (ifenprodil vs. sham: **p = 0.0015; HIBD vs. sham, *p = 0.0290; HIBD vs. ifenprodil, *p = 0.0479; NaCl vs. sham, **p = 0.0013; NaCl vs. ifenprodil, *p = 0.0113, *n* = 3)), indicating that overactivation of the PERK-eIF2α signaling pathway leads to more neuronal death. Hoechst staining results after administering CCT020312 (a selective PERK/eIF2α agonist) post-HI to activate the PERK-eIF2α signaling pathway are consistent with this Figure 5A, suggesting that in post-HI, the highly expressed GluN2B receptor exerts neuroprotective effects by inhibiting the overactivation of the PERK-eIF2α signaling pathway through the PI3K/AKT pathway.

### 3.4 Ifenprodil exacerbates tissue pathology and neuronal apoptosis

Brain tissue slices from control, HIBD, and ifenprodil groups were examined. HE staining revealed that ifenprodil further increased the area of cavities appearing post-modeling and led to looser tissue arrangement, which is detrimental to neuronal migration and cortical development. Additionally, Nissl staining indicated a reduction in the number of cortical neurons in HIBD mice, with granular pathological changes in Nissl bodies, and further reduction in neuronal numbers post-ifenprodil administration (Figure 5B). This suggests neuronal apoptosis and a decrease in the number of neurons, consistent with the above research findings.

### 3.5 Ifenprodil and CCT020312 further impairs spatial memory and emotions in HIBD mice

**Figure 6:**
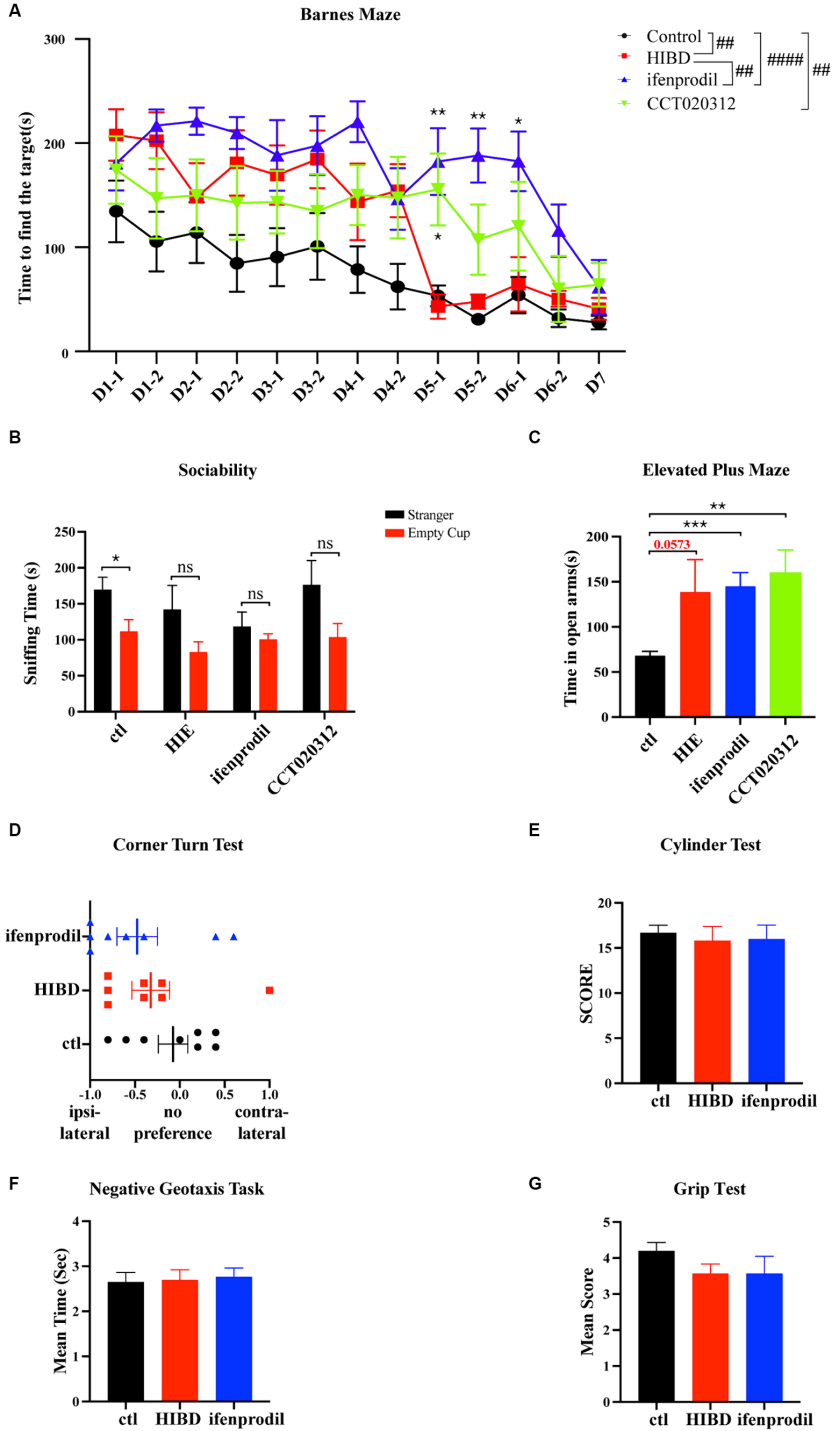
Ifenprodil and CCT020312 aggravates deficits in spatial memory, cognition, and social abilities in mice. **(A)** HIE, ifenprodil and CCT020312 group mice after surgery sent back to the original cages until they were 4 weeks old, and control mice were the same age. The Barnes maze test was used to detected the spatial memory of control, HIE, ifenprodil and CCT020312 group mice. #indicates overall differences in performance of control, HIE and ifenprodil group mice (##*p* < 0.01, ####*p* < 0.0001, *n* = 8); ^*^indicates that compared with HIBD group, differences of ifenprodil and CCT020312 group at the same time point (^*^p < 0.05, ^**^p < 0.01, *n* = 8). **(B)** The three-chamber social interaction test was used to detected the sociability of control, HIE, ifenprodil and CCT020312 group mice. ^*^indicates that differences between sniffing time of stranger and that of empty cup (*p < 0.05, *n* = 8). **(C)** The elevated plus maze was used to detect the mood of control, HIE, ifenprodil and CCT020312 group mice. *indicates that differences between time spent in the open arms of ifenprodil and that of control (^**^p < 0.01, ^***^p < 0.001, *n* = 8). **(D–G)** The corner turn test, cylinder test, negative geotaxis task and grip test were used to detect the motor function.

Considering the weak physique and poor mental state of the mice in the HIBD and ifenprodil groups at 4 weeks of age, the Barnes maze test was used to assess their spatial memory capabilities. Overall, the model group mice took longer to find the target hole compared to the control group, and this time was further extended after ifenprodil treatment and CCT020312 treatment (Overall differences: control group vs. HIE group: #*p* = 0.0133; control group vs. ifenprodil group: ####*p* < 0.0001; HIE group vs. ifenprodil group: #*p* = 0.0128; control group vs. CCT020312 group: ##*p* = 0.0038), especially noticeable on training days 5 and 6 (Training D5-1: ifenprodil group vs. HIBD group: **p = 0.0015, CCT020312 vs. HIBD group: *p = 0.0216; training D5-2: ifenprodil group vs. HIBD group: **p = 0.0013; training D6-1: ifenprodil group vs. HIBD group: *p = 0.0103). This suggests that inhibiting GluN2B or activating PERK-eIF2α pathway further impairs the spatial memory of HIBD mice (Figure 6A). During the three-chamber social interaction test, control group mice spent more time sniffing the stranger mouse, indicating strong social interest, while HIBD group, ifenprodil group and CCT020312 group mice showed equal exploration time for the empty cup and stranger mouse, indicating reduced social desire, possibly related to cognitive impairment (Sniffing time of control group: stranger vs. empty cup: *p = 0.0296, n = 8; sniffing time of HIBD group: stranger vs. empty cup: *p* = 0.1245, *n* = 8; sniffing time of ifenprodil group: stranger vs. empty cup: *p* = 0.4204, *n* = 8; sniffing time of CCT020312 group: stranger vs. empty cup: *p* = 0.0848, *n* = 7) (Figure 6B). HIBD group, ifenprodilgroup and CCT020312 group mice mice spent more time in the open arms of the elevated plus maze, reflecting a lower fear response to the external environment, demonstrating a “fearless due to ignorance” state (Time in the open arms: ifenprodil vs. control: ***p = 0.0003; HIBD vs. control: *p* = 0.0573; CCT020312 vs. Control: **p = 0.0026) (Figure 6C). Further motor function tests such as the corner turn test, cylinder test, negative geotaxis task, and Grip Test did not reveal significant differences (Score of corner turn test: HIBD vs. control: *p* = 0.3648; ifenprodil vs. control: *p* = 0.1750; ifenprodil vs. HIBD: *p* = 0.6350, *n* = 8) (Score of cylinder test: HIBD vs. control: *p* = 0.6292; ifenprodil vs. control: *p* = 0.7007; ifenprodil vs. HIBD: *p* = 0.9332, *n* = 8) (The mean time of negative geotaxis task: HIBD vs. control: *p* = 0.8859; ifenprodil vs. control: *p* = 0.6865; ifenprodil vs. HIBD: *p* = 0.8105, *n* = 8) (Score of grip test: HIBD vs. control: *p* = 0.0953; ifenprodil vs. control: *p* = 0.2589; ifenprodil vs. HIBD: *p* > 0.9999, *n* = 8) (Figures 6D–G). However, in the corner turn test, both HIBD and ifenprodil-treated mice showed a preference for turning to one side (Figure 6D). These results indicate that intraperitoneal injection ifenprodil to limit the function of GluN2B further decreases the spatial memory, cognitive levels, and social abilities of mice. After injecting intraperitoneally with CCT020312 to activate PERK/eIF2α pathway, the mice also showed the same damage. But there is no difference in motor functions among the different groups.

## 4 Discussion

Numerous studies have suggested that excessive activation of NMDA receptors (NMDARs) is a key factor in ischemic brain damage in adults (Luo et al., 2018). However, NMDAR antagonists have not been very effective in clinical treatment of ischemic injury (Ikonomidou and Turski, 2002). This may be because NMDARs are involved in many important brain activities, such as dendritic spine formation and brain maturation (Cull-Candy et al., 2001; Lai et al., 2014; Hardingham, 2019), and the use of selective NMDAR antagonists can cause various adverse reactions, such as cognitive impairment and hallucinations (Kemp and McKernan, 2002; Lipton, 2006). This is related to the “double-edged sword” effect of NMDARs, where the activation of NMDARs is associated with changes in the activity of many pro-apoptotic and pro-survival transcription factors (West et al., 2002; Hetman and Kharebava, 2006; Zhang et al., 2007). The “localization hypothesis” suggests that the activation of NMDARs located on dendritic spines promotes cell survival, while the activation of extrasynaptic NMDARs promotes cell death (Hardingham et al., 2002). Although it was previously thought that this was due to different subunits in synaptic and extrasynaptic NMDARs, this idea has been refuted by numerous studies, as different subunits are distributed both inside and outside the cell (Thomas et al., 2006; Harris and Pettit, 2007; Sanz-Clemente et al., 2013). According to the “subunit hypothesis,” GluN2A-type NMDARs are more generally associated with cell survival, while GluN2B-type NMDARs are associated with cell death signaling (Cepeda and Levine, 2012). However, it seems more appropriate to consider a “unified hypothesis,” where extrasynaptic GluN2B-NMDAR activation sends death signals to the cell faster than synaptic GluN2A-NMDAR (Lai et al., 2011). Thus, in stroke models, the use of GluN2B inhibitors such as ifenprodil, its derivatives, and Tat-NR2B9c peptide can effectively reduce the area of brain infarction (Gotti et al., 1988; Cook et al., 2012). These hypotheses seem more applicable to mature neurons, as in our previous research, dominant GluN2B in immature neurons exhibited neuroprotective effects in neonatal hypoxic–ischemic brain damage, and ifenprodil exacerbated damage by further inhibiting the PI3K/AKT signaling pathway, which is different from its behavior in adults (Zhang et al., 2023). This may be due to the imbalance in the expression of NMDAR subunits during development, where GluN2B is expressed much higher than GluN2A in early stages, playing a protective role, but this is gradually reversed as development progresses. However, how this transformation occurs, whether it simply depends on the difference in expression, and whether there are specific time points are unclear. Our study results suggest that treatment methods for mature neurons are not entirely suitable for hypoxic–ischemic injury in immature neurons, hence the urgent need to explore its mechanisms and find more suitable treatment targets.

The pathogenesis of neonatal hypoxic–ischemic brain damage (HIBD) is complex, involving multiple pathological processes such as energy depletion, oxidative stress, excitotoxicity, and mitochondrial damage. Hypoxic–ischemic events lead to insufficient oxygen supply and ATP exhaustion (Rodriguez et al., 2020). The endoplasmic reticulum (ER) is responsible for the synthesis and transport of secretory proteins and lipids within the cell, requiring a large amount of ATP to maintain normal function (Depaoli et al., 2019). The occurrence of hypoxia-ischemia cuts off energy supply, leading to ER dysfunction, an accumulation of peptide chains and proteins in the ER, and triggering an ER stress response. To restore normal function, the downstream unfolded protein response (UPR) pathway is activated, helping to alleviate ER load in various ways (Hetz et al., 2020). Current studies on UPR in the pathogenesis of HIBD are limited, and it is unclear which of the three pathways plays a major role (Badiola et al., 2011; Carloni et al., 2014; Chavez-Valdez et al., 2016; Huang et al., 2020). We selected four time points post-HI (3 h, 6 h, 24 h, 48 h) for investigation and found that the PERK/eIF2α pathway showed the strongest response. IRE1 pathway exhibited a transient downregulation only at 6 h, so we focused on the PERK/eIF2α pathway for further study. The phosphorylation level of eIF2α increased indicate that the PERK/eIF2α pathway may be activated. Prolonged activation of the PERK/eIF2α pathway did not restore ER homeostasis and resist apoptosis as expected, but instead, excessive activation mediated apoptosis, reflected in increased expression of cleaved caspase3 (Figure 2).

We wanted to know whether the different neuroprotective role of GluN2B in immature neurons compared to mature neurons was related to the UPR pathway. We chose 48 h post-HI, the most severe stage of death, for analysis. Compared to the HIBD group, ifenprodil-treated mice had higher levels of phosphorylation of PERK and eIF2α, and increased expression of CHOP and Cleaved Caspase3, suggesting a more intense reaction of the PERK/eIF2α pathway at this time, and this excessive activation led to more severe death (Figure 4). HE staining indicated that after inhibiting GluN2B with ifenprodil, the cortical tissue showed more severe pathological morphology, with a looser arrangement and more obvious vacuolation, obviously unable to maintain normal brain morphology and neuronal growth and migration. Nissl staining reflected a significant reduction in the number of neurons and granular changes in Nissl bodies, consistent with the findings that inhibition of the dominant subunit GluN2B unbound the PERK/eIF2α pathway, leading to more severe death in an excessively activated state (Figure 5). Severe tissue pathology was also reflected in behavior; four-week-old mice treated with ifenprodil showed varying degrees of impairment in spatial memory, cognitive level, and social abilities. In consistent with injection ifenprodil, overactivating PERK/eIF2ɑ pathway showed the same impairment (Figure 5). If reflected in HIBD patients, such loss of abilities essential for normal life would undoubtedly bring a heavy burden to families and society. This also indicates that in early development, the role of GluN2B is crucial (Wang et al., 2011).

Previous research has proven that the pathogenesis of neonatal hypoxic–ischemic brain damage involves three stages: primary cellular injury, energy restoration during resuscitation, and delayed cellular injury, with effective intervention within 6 h of birth in HIBD patients being more meaningful (Garcia-Alix et al., 2023). According to our study, a transient downregulation in the expression of the three branches of the UPR pathway occurs at 6 h, followed by continuous activation of the PERK/eIF2α pathway leading to irreversible brain damage. Is the special phenomenon at 6 h a key point where the UPR pathway shifts from protective to lethal effects? Could limiting the activation of the UPR pathway to a reasonable level after six hours prolong its protective effect? This might provide new insights for treating HIBD. Currently, hypothermia therapy is the only approved treatment method, but its high cost and uncertain prognostic effects remain a concern (Ranjan and Gulati, 2023; Thayyil et al., 2023). Treatment starting within 6 h shows neuroprotective effects, posing a huge challenge for early diagnosis of HIBD. The absence of reliable indicators to reflect the prognosis after the end of the 72-h continuous treatment is also an urgent problem to be solved (Garcia-Alix et al., 2023). The lack of good therapeutic effects in mild HIBD patients (Ehlting et al., 2022), differences in treatment effects between male and female rats (Saadat et al., 2023), and other instabilities are driving research into the pathogenesis of HIBD, with the hope of proposing new targets to supplement and compensate for the shortcomings of hypothermia therapy. Our study elucidates that GluN2B plays a neuroprotective role in early development by inhibiting excessive activation of the PERK/eIF2α pathway. One deficiency of this work is that although Ifenprodil is a selective GluN2B-NMDAR antagonist that has shown neuroprotective effects in a variety of adult ischemic encephalopathy through inhibiting GluN2B-NMDAR (Gotti et al., 1988; Chen et al., 2008; Gutierrez-Vargas et al., 2014), still we noticed that some studies have shown that ifenprodil can interact with a variety of other receptors, such as GIRK and α1 adrenergic receptors (Kobayashi et al., 2006). Therefore, we could not exclude the potential effects of these receptors in HIBD which is interesting and worth further study.

In summary, in this study we verified that GluN2B-NMDAR plays a neuroprotective role in the pathogenesis of HIBD, which is different from GluN2B-NMDAR mediated death in mature neurons. And this study suggested a potential role GluN2B-NMDAR in balancing the activation state of the UPR signaling pathway, thereby regulating the transition between pro-survival and pro-apoptosis. Our study may provide new ideas for further explore potential treatment target for HIBD: (1) Activation of GluN2B-NMDAR by NMDA administration to play a role in neuroprotective effects; (2) Searching for the signaling pathway between GluN2B NMDAR and UPR and using it as a target for intervention; (3) Targeting key proteins in the UPR pathway to regulate the activity status of the UPR signaling pathway.

## Data availability statement

The original contributions presented in the study are included in the article/supplementary material, further inquiries can be directed to the corresponding author.

## Ethics statement

The animal study was approved by The Animal Care and Use Committee of Kunming Medical University. The study was conducted in accordance with the local legislation and institutional requirements.

## Author contributions

MW: Writing – original draft, Writing – review & editing, Investigation. SX: Writing – review & editing, Investigation, Funding acquisition. KM: Writing – review & editing, Investigation. SY: Writing – review & editing, Investigation. YX: Writing – review & editing, Investigation. JL: Writing – review & editing, Investigation. JC: Writing – review & editing, Investigation. XZ: Writing – original draft, Writing – review & editing, Conceptualization, Resources, Supervision, Funding acquisition.

## References

[ref1] Badiola N. Penas C. Minano-Molina A. Barneda-Zahonero B. Fado R. Sanchez-Opazo G.. (2011). Induction of ER stress in response to oxygen-glucose deprivation of cortical cultures involves the activation of the PERK and IRE-1 pathways and of caspase-12. Cell Death Dis. 2:e149. doi: 10.1038/cddis.2011.31, PMID: 21525936 PMC3122062

[ref2] Brittain M. K. Brustovetsky T. Brittain J. M. Khanna R. Cummins T. R. Brustovetsky N. (2012). Ifenprodil, a NR2B-selective antagonist of NMDA receptor, inhibits reverse Na+/Ca2+ exchanger in neurons. Neuropharmacology 63, 974–982. doi: 10.1016/j.neuropharm.2012.07.012, PMID: 22820271 PMC3427421

[ref3] Carloni S. Albertini M. C. Galluzzi L. Buonocore G. Proietti F. Balduini W. (2014). Melatonin reduces endoplasmic reticulum stress and preserves sirtuin 1 expression in neuronal cells of newborn rats after hypoxia-ischemia. J. Pineal Res. 57, 192–199. doi: 10.1111/jpi.12156, PMID: 24980917

[ref4] Cepeda C. Levine M. S. (2012). 2B or not 2B: a tail of two NMDA receptor subunits. Neuron 74, 426–428. doi: 10.1016/j.neuron.2012.04.011 22578493

[ref5] Chavez-Valdez R. Flock D. L. Martin L. J. Northington F. J. (2016). Endoplasmic reticulum pathology and stress response in neurons precede programmed necrosis after neonatal hypoxia-ischemia. Int. J. Dev. Neurosci. 48, 58–70. doi: 10.1016/j.ijdevneu.2015.11.007, PMID: 26643212 PMC4718855

[ref6] Chen M. Lu T. J. Chen X. J. Zhou Y. Chen Q. Feng X. Y.. (2008). Differential roles of NMDA receptor subtypes in ischemic neuronal cell death and ischemic tolerance. Stroke 39, 3042–3048. doi: 10.1161/STROKEAHA.108.521898, PMID: 18688011

[ref7] Cook D. J. Teves L. Tymianski M. (2012). Treatment of stroke with a PSD-95 inhibitor in the gyrencephalic primate brain. Nature 483, 213–217. doi: 10.1038/nature10841, PMID: 22388811

[ref8] Cull-Candy S. Brickley S. Farrant M. (2001). NMDA receptor subunits: diversity, development and disease. Curr. Opin. Neurobiol. 11, 327–335. doi: 10.1016/S0959-4388(00)00215-4 11399431

[ref9] Depaoli M. R. Hay J. C. Graier W. F. Malli R. (2019). The enigmatic ATP supply of the endoplasmic reticulum. Biol. Rev. Camb. Philos. Soc. 94, 610–628. doi: 10.1111/brv.12469, PMID: 30338910 PMC6446729

[ref10] Ehlting A. Zweyer M. Maes E. Schleehuber Y. Doshi H. Sabir H.. (2022). Impact of hypoxia-ischemia on neurogenesis and structural and functional outcomes in a mild-moderate neonatal hypoxia-ischemia brain injury model. Life 12:1164. doi: 10.3390/life12081164 36013343 PMC9410039

[ref11] Garcia-Alix A. Arnaez J. Herranz-Rubia N. Alarcon A. Arca G. Valverde E.. (2023). Ten years since the introduction of therapeutic hypothermia in neonates with perinatal hypoxic-ischaemic encephalopathy in Spain. Neurologia 38, 364–371. doi: 10.1016/j.nrl.2020.05.017, PMID: 35260363

[ref12] Glass H. C. Shellhaas R. A. Wusthoff C. J. Chang T. Abend N. S. Chu C. J.. (2016). Contemporary profile of seizures in neonates: a prospective cohort study. J Pediatr 174, 98–103.e1. doi: 10.1016/j.jpeds.2016.03.035, PMID: 27106855 PMC4925241

[ref13] Gotti B. Duverger D. Bertin J. Carter C. Dupont R. Frost J.. (1988). Ifenprodil and SL 82.0715 as cerebral anti-ischemic agents. I. Evidence for efficacy in models of focal cerebral ischemia. J. Pharmacol. Exp. Ther. 247, 1211–1221. PMID: 2849668

[ref14] Gutierrez-Vargas J. A. Munoz-Manco J. I. Garcia-Segura L. M. Cardona-Gomez G. P. (2014). Glu N2B N-methyl-D-aspartic acid receptor subunit mediates atorvastatin-induced neuroprotection after focal cerebral ischemia. J. Neurosci. Res. 92, 1529–1548. doi: 10.1002/jnr.23426, PMID: 24939000

[ref15] Hardingham G. (2019). NMDA receptor C-terminal signaling in development, plasticity, and disease. F1000Res 8:1547. doi: 10.12688/f1000research.19925.1 PMC6720038 31508206

[ref16] Hardingham G. E. Fukunaga Y. Bading H. (2002). Extrasynaptic NMDARs oppose synaptic NMDARs by triggering CREB shut-off and cell death pathways. Nat. Neurosci. 5, 405–414. doi: 10.1038/nn835, PMID: 11953750

[ref17] Harris A. Z. Pettit D. L. (2007). Extrasynaptic and synaptic NMDA receptors form stable and uniform pools in rat hippocampal slices. J. Physiol. 584, 509–519. doi: 10.1113/jphysiol.2007.137679 17717018 PMC2277145

[ref18] Hetman M. Kharebava G. (2006). Survival signaling pathways activated by NMDA receptors. Curr. Top. Med. Chem. 6, 787–799. doi: 10.2174/156802606777057553 16719817

[ref19] Hetz C. Zhang K. Kaufman R. J. (2020). Mechanisms, regulation and functions of the unfolded protein response. Nat. Rev. Mol. Cell Biol. 21, 421–438. doi: 10.1038/s41580-020-0250-z, PMID: 32457508 PMC8867924

[ref20] Huang J. Lu W. Doycheva D. M. Gamdzyk M. Hu X. Liu R.. (2020). IRE1alpha inhibition attenuates neuronal pyroptosis via mi R-125/NLRP1 pathway in a neonatal hypoxic-ischemic encephalopathy rat model. J. Neuroinflammation 17:152. doi: 10.1186/s12974-020-01796-3, PMID: 32375838 PMC7203836

[ref21] Ikonomidou C. Turski L. (2002). Why did NMDA receptor antagonists fail clinical trials for stroke and traumatic brain injury? Lancet Neurol. 1, 383–386. doi: 10.1016/S1474-4422(02)00164-3 12849400

[ref22] Jia W. Lei X. Dong W. Li Q. (2018). Benefits of starting hypothermia treatment within 6 h vs. 6-12 h in newborns with moderate neonatal hypoxic-ischemic encephalopathy. BMC Pediatr. 18:50. doi: 10.1186/s12887-018-1013-2, PMID: 29433475 PMC5809807

[ref23] Karadottir R. Cavelier P. Bergersen L. H. Attwell D. (2005). NMDA receptors are expressed in oligodendrocytes and activated in ischaemia. Nature 438, 1162–1166. doi: 10.1038/nature04302, PMID: 16372011 PMC1416283

[ref24] Kemp J. A. McKernan R. M. (2002). NMDA receptor pathways as drug targets. Nat. Neurosci. 5, 1039–1042. doi: 10.1038/nn936 12403981

[ref25] Knox R. Zhao C. Miguel-Perez D. Wang S. Yuan J. Ferriero D.. (2013). Enhanced NMDA receptor tyrosine phosphorylation and increased brain injury following neonatal hypoxia-ischemia in mice with neuronal Fyn overexpression. Neurobiol. Dis. 51, 113–119. doi: 10.1016/j.nbd.2012.10.024, PMID: 23127881 PMC3595007

[ref26] Kobayashi T. Washiyama K. Ikeda K. (2006). Inhibition of G protein-activated inwardly rectifying K+ channels by ifenprodil. Neuropsychopharmacology 31, 516–524. doi: 10.1038/sj.npp.1300844 16123769

[ref27] Lai Q. Hu P. Li Q. Li X. Yuan R. Tang X.. (2016). NMDA receptors promote neurogenesis in the neonatal rat subventricular zone following hypoxic-ischemic injury. Mol. Med. Rep. 13, 206–212. doi: 10.3892/mmr.2015.4501, PMID: 26548659 PMC4686072

[ref28] Lai T. W. Shyu W. C. Wang Y. T. (2011). Stroke intervention pathways: NMDA receptors and beyond. Trends Mol. Med. 17, 266–275. doi: 10.1016/j.molmed.2010.12.008, PMID: 21310659

[ref29] Lai T. W. Zhang S. Wang Y. T. (2014). Excitotoxicity and stroke: identifying novel targets for neuroprotection. Prog. Neurobiol. 115, 157–188. doi: 10.1016/j.pneurobio.2013.11.006, PMID: 24361499

[ref30] Lee A. C. Kozuki N. Blencowe H. Vos T. Bahalim A. Darmstadt G. L.. (2013). Intrapartum-related neonatal encephalopathy incidence and impairment at regional and global levels for 2010 with trends from 1990. Pediatr. Res. 74, 50–72. doi: 10.1038/pr.2013.206, PMID: 24366463 PMC3873711

[ref31] Lipton S. A. (2006). Paradigm shift in neuroprotection by NMDA receptor blockade: memantine and beyond. Nat. Rev. Drug Discov. 5, 160–170. doi: 10.1038/nrd1958, PMID: 16424917

[ref32] Luo Y. Ma H. Zhou J. J. Li L. Chen S. R. Zhang J.. (2018). Focal cerebral ischemia and reperfusion induce brain injury through alpha 2delta-1-bound NMDA receptors. Stroke 49, 2464–2472. doi: 10.1161/STROKEAHA.118.022330, PMID: 30355118 PMC6205748

[ref33] Luo J. Wang Y. Yasuda R. P. Dunah A. W. Wolfe B. B. (1997). The majority of N-methyl-D-aspartate receptor complexes in adult rat cerebral cortex contain at least three different subunits (NR1/NR2A/NR2B). Mol. Pharmacol. 51, 79–86. doi: 10.1124/mol.51.1.79, PMID: 9016349

[ref34] Martel M. A. Wyllie D. J. Hardingham G. E. (2009). In developing hippocampal neurons, NR2B-containing N-methyl-D-aspartate receptors (NMDARs) can mediate signaling to neuronal survival and synaptic potentiation, as well as neuronal death. Neuroscience 158, 334–343. doi: 10.1016/j.neuroscience.2008.01.080, PMID: 18378405 PMC2635533

[ref35] Nabetani M. Mukai T. Shintaku H. (2022). Preventing brain damage from hypoxic-ischemic encephalopathy in neonates: update on mesenchymal stromal cells and umbilical cord blood cells. Am. J. Perinatol. 39, 1754–1763. doi: 10.1055/s-0041-1726451, PMID: 33853147 PMC9674406

[ref36] Ranjan A. K. Gulati A. (2023). Advances in therapies to treat neonatal hypoxic-ischemic encephalopathy. J. Clin. Med. 12:6653. doi: 10.3390/jcm12206653, PMID: 37892791 PMC10607511

[ref37] Rauner C. Kohr G. (2011). Triheteromeric NR1/NR2A/NR2B receptors constitute the major N-methyl-D-aspartate receptor population in adult hippocampal synapses. J. Biol. Chem. 286, 7558–7566. doi: 10.1074/jbc.M110.182600, PMID: 21190942 PMC3045010

[ref38] Rodriguez M. Valez V. Cimarra C. Blasina F. Radi R. (2020). Hypoxic-ischemic encephalopathy and mitochondrial dysfunction: facts, unknowns, and challenges. Antioxid. Redox Signal. 33, 247–262. doi: 10.1089/ars.2020.8093, PMID: 32295425

[ref39] Saadat A. Blackwell A. Kaszowski C. Pallera H. Owens D. Lattanzio F.. (2023). Therapeutic hypothermia demonstrates sex-dependent improvements in motor function in a rat model of neonatal hypoxic ischemic encephalopathy. Behav. Brain Res. 437:114119. doi: 10.1016/j.bbr.2022.114119 36162642

[ref40] Sanz-Clemente A. Nicoll R. A. Roche K. W. (2013). Diversity in NMDA receptor composition: many regulators, many consequences. Neuroscientist 19, 62–75. doi: 10.1177/1073858411435129, PMID: 22343826 PMC3567917

[ref41] Siu C. R. Beshara S. P. Jones D. G. Murphy K. M. (2017). Development of glutamatergic proteins in human visual cortex across the lifespan. J. Neurosci. 37, 6031–6042. doi: 10.1523/JNEUROSCI.2304-16.2017, PMID: 28554889 PMC6596503

[ref42] Stockwell S. R. Platt G. Barrie S. E. Zoumpoulidou G. Te Poele R. H. Aherne G. W.. (2012). Mechanism-based screen for G1/S checkpoint activators identifies a selective activator of EIF2ɑK3/PERK signalling. PLoS One 7:e28568. doi: 10.1371/journal.pone.0028568, PMID: 22253692 PMC3257223

[ref43] Takagi N. Sasakawa K. Besshoh S. Miyake-Takagi K. Takeo S. (2003). Transient ischemia enhances tyrosine phosphorylation and binding of the NMDA receptor to the Src homology 2 domain of phosphatidylinositol 3-kinase in the rat hippocampus. J. Neurochem. 84, 67–76. doi: 10.1046/j.1471-4159.2003.01500.x, PMID: 12485402

[ref44] Thayyil S. Montaldo P. Krishnan V. Ivain P. Pant S. Lally P. J.. (2023). Whole-body hypothermia, cerebral magnetic resonance biomarkers, and outcomes in neonates with moderate or severe hypoxic-ischemic encephalopathy born at tertiary care Centers vs other facilities: a nested study within a randomized clinical trial. JAMA Netw. Open 6:e2312152. doi: 10.1001/jamanetworkopen.2023.12152, PMID: 37155168 PMC10167567

[ref45] Thomas C. G. Miller A. J. Westbrook G. L. (2006). Synaptic and extrasynaptic NMDA receptor NR2 subunits in cultured hippocampal neurons. J. Neurophysiol. 95, 1727–1734. doi: 10.1152/jn.00771.2005, PMID: 16319212

[ref46] Traynelis S. F. Wollmuth L. P. McBain C. J. Menniti F. S. Vance K. M. Ogden K. K.. (2010). Glutamate receptor ion channels: structure, regulation, and function. Pharmacol. Rev. 62, 405–496. doi: 10.1124/pr.109.002451, PMID: 20716669 PMC2964903

[ref47] Wang C. C. Held R. G. Chang S. C. Yang L. Delpire E. Ghosh A.. (2011). A critical role for Glu N2B-containing NMDA receptors in cortical development and function. Neuron 72, 789–805. doi: 10.1016/j.neuron.2011.09.023, PMID: 22153375

[ref48] West A. E. Griffith E. C. Greenberg M. E. (2002). Regulation of transcription factors by neuronal activity. Nat. Rev. Neurosci. 3, 921–931. doi: 10.1038/nrn987 12461549

[ref49] Wu Q. J. Tymianski M. (2018). Targeting NMDA receptors in stroke: new hope in neuroprotection. Mol. Brain 11:15. doi: 10.1186/s13041-018-0357-8, PMID: 29534733 PMC5851248

[ref50] Zhang X. T. Peng K. Z. Xu S. L. Wu M. X. Sun H. J. Zhao J.. (2023). The Glu N2B-containing NMDA receptor alleviates neuronal apoptosis in neonatal hypoxic-ischemic encephalopathy by activating PI3K-Akt-CREB Signaling pathway. Physiol. Res. 72, 669–680. doi: 10.33549/physiolres.935044, PMID: 38015765 PMC10751047

[ref51] Zhang S. J. Steijaert M. N. Lau D. Schutz G. Delucinge-Vivier C. Descombes P.. (2007). Decoding NMDA receptor signaling: identification of genomic programs specifying neuronal survival and death. Neuron 53, 549–562. doi: 10.1016/j.neuron.2007.01.025, PMID: 17296556

